# The Interplay of Four Main Pathways Recomposes Immune Landscape in Primary and Metastatic Gastroenteropancreatic Neuroendocrine Tumors

**DOI:** 10.3389/fonc.2022.808448

**Published:** 2022-05-18

**Authors:** Xin Lou, Heli Gao, Xiaowu Xu, Zeng Ye, Wuhu Zhang, Fei Wang, Jie Chen, Yue Zhang, Xuemin Chen, Yi Qin, Xianjun Yu, Shunrong Ji

**Affiliations:** ^1^Center for Neuroendocrine Tumors, Fudan University Shanghai Cancer Center, Shanghai, China; ^2^Department of Pancreatic Surgery, Fudan University Shanghai Cancer Center, Shanghai, China; ^3^Department of Oncology, Shanghai Medical College, Fudan University, Shanghai, China; ^4^Shanghai Pancreatic Cancer Institute, Shanghai, China; ^5^Pancreatic Cancer Institute, Fudan University, Shanghai, China; ^6^Department of Hepatopancreatobiliary Surgery, the Third Affiliated Hospital of Soochow University, Changzhou, China

**Keywords:** gastroenteropancreatic neuroendocrine neoplasms, chromosomal instability, telomere maintenance, MTOR signaling, DNA damage repair, immune landscape

## Abstract

**Background:**

The four major pathways in gastroenteropancreatic neuroendocrine neoplasms (GEP-NENs) including chromatin remodeling, DNA damage repair, activation of mTOR signaling, and telomere maintenance were mediated by some critical molecules and constituted critical processes of regulation in cancer-causing processes. However, the interplay and potential role of these pathway-related molecules in the tumor microenvironment of the primary and metastatic site remained unknown.

**Methods:**

We systematically evaluated the mRNA expression of 34 molecules associated with the four pathways in 227 GEP−NEN samples from 5 datasets. We assigned the samples into two expression patterns of pathway-related molecules by an unsupervised clustering method. Subsequently, we explored the specific cell-related molecules, especially immune and stromal cells using the WGCNA method, based on differentially expressed genes (DEGs) responsible for the different patterns of pathway-related molecules, which provided a new method to qualify the pathway-related subtypes of individual tumors, then the PC_Score and PI_Score scoring systems were also constructed using obtained specific cell-related molecules. Furthermore, we performed the association of pathway-related subtypes with characteristics of immune landscape in primary and metastatic GEP-NENs.

**Results:**

We demonstrated that the specific pathway-related molecules (SMARCA4, MLH1, TSC1, ATRX, and ATR) were associated with cytolytic activity. Then we identified the two distinct patterns of pathway-related molecules, which were characteristic with a significantly distinct immune landscape. Using WGCNA, we also identified the fibroblast-related molecules, including ASPN, COL10A1, COL3A1, EDNRA, MYL9, PRELP, RAB31, SPARC, and THBS2, and immune-related molecules including CASP1, CCL5, CTSS, CYBRD1, PMP22, and TFEC. Based on these specific markers, we identified four distinct pathway-related subtypes, characterized by immune and fibrotic enriched (I/FE), immune enriched (IE), fibrotic enriched (FE), and immune and fibrotic desert (I/FD), of which I/FE was characteristic with the highest PC_Score and PI_Score whereas I/FD presents the opposite trend. I/FE was positively correlated with immune landscape of T-cell activation and immunosuppression. Furthermore, the I/FE marked GEP-NENs with increased immune activation scores (T-cell costimulation, MHC I presentation, and APC costimulation). Importantly, the four distinct pathway-related subtypes were not conserved in different tumor sites, because I/FE was lacking in the liver metastatic site even though IE, FE, and I/FD also could be observed in the metastatic site.

**Conclusions:**

This study was the first to perform a comprehensive analysis of the four major pathways in GEP-NENs. We demonstrated the potential function of these pathway-related molecules in immune landscapes. Our findings indicated that the primary and metastatic GEP-NENs had distinct antitumor phenotypes. This work highlighted the interplay and potential clinical utility of these pathway-related molecules in GEP-NENs.

## Introduction

Gastroenteropancreatic neuroendocrine neoplasms (GEP−NENs), a rare tumor, accounted for about 1% of all malignancies and were the second most common form of gastrointestinal malignancy with a mortality rate of 60% (1). With a few improvements in treatment or survival, the development of new treatment strategies was urgently needed. Immunotherapy had brought the huge benefit in most of common tumors but had remained ineffective in GEP-NENs (2). One of the reasons for this was our insufficient knowledge of the molecular functions which might have a critical role in the formation of antitumor immune landscapes in GEP-NENs (3).

Currently, the prerequisites for good immunotherapy were the preexistence of immune checkpoints and the higher infiltration of effector CD8 T cells (4). The previous study confirmed that the higher tumor mutation burden and genomic instability had a positive correlation with the CD8 T-cell infiltration level and immune response (5). Notably, GEP-NENs had a lower tumor mutation burden and relative-simplicity genomes, being driven by chromosomal instability, telomere maintenance, and other biological processes (6), which might be the critical reason for the worse response to immune therapy (2). However, a small proportion of GEP-NEN patients was characterized with a higher CD8 T-cell infiltration which was clinically favorable (7), which reflected the presence of drivers of antitumor immunity in GEP-NENs.

Plenty of studies had pointed that the mutations of GEP-NENs, including point mutations and gene fusions, had been found in molecules associated with four main pathways: chromatin remodeling, DNA damage repair, activation of mTOR signaling, and telomere maintenance (6, 8, 9). However, the role of these molecules in mounting an immune activation in GEP-NENs has not been explored. Here, we emphasized the expression of a distinct pathway-related signatures (SMARCA4, MLH1, TSC1, ATRX, ATR) that might play a critical role in the formation of antitumor immune landscape in GEP-NENs. Then, based on the pathway-related genes, we also calculated the specific cell-related molecules, reclassified GEP-NEN samples into four subgroups (I/FE, IE, FE, I/FD), and established a new PI_Score and PC_Score scoring model. These findings might assist the identification of GEP-NEN samples who could benefit from immunotherapy.

## Method

### Data Collection and Processing

Public gene expression data were retrieved in Gene Expression Omnibus (GEO). Five GEO cohorts (GSE43797, GSE65286, GSE73338, GSE73339, GSE117851) were used as discovery groups which include 204 GEP-NENs and 23 normal samples while another cohort (GSE98894) was set as the validated group including 130 primary samples and 69 liver metastases. The batch effects from non-biotechnological bias was corrected by the “sva” package (10), an R Bioconductor package. The data were analyzed using R (version 4.1.1)

### Clustering Expression Pattern of Pathway-Related Molecules

The specific pathway-related molecules associated with GEP-NENs include 11 telomere maintenance (DAXX, ATRX, TERF1, ATR, SMC5, SMC6, RAD52, RAD51AP1) (11), 5 chromatin remodeling (SETD2, YY1, SMARCA4) (12), 9 mTOR signaling (PTEN, TSC1, TSC2, DEPDC5, PSPN EWSR1, PIK3CA, AKT1) (6), and 15 DNA damage repair (CHEK2, BRCA2, MUTYH, ATM, ERCC1, BRCA1, MLH3, MLH1, MSH2, MSH3, MSH4, MSH6, PMS1, MGMT, MEN1) (13). More detail is available in **Table S1**. Then, we explore the relationship of pathway-related molecules with the immune infiltration levels. Cytolytic activity (CYT) was defined as the geometric mean of the RNA-seq expression of GZMA and PRF1 in tumor tissue (14). Based on the significant pathway-related genes, unsupervised clustering was used to identify the robust clustering of GEP-NENs, which was achieved using the “Consensus-Clusterplus” package of R, and we also conducted 1,000 repetitions to validate stability of the classification (15).

### Identification of DEGs Between Pathways Distinct Phenotypes

We classified all GEP-NNE samples into two distinct subtypes based on the expression of pathway-related molecules. The empirical Bayesian approach of the “limma” R package was used to identify DEGs between different pathway-related patterns (16), with the threshold of adjusted P value < 0.05 and |logFC|>0.5.

### Gene Set Variation Analysis and Functional Annotation

We conducted gene set variation analysis (GSVA) enrichment analysis using “GSVA” R packages to investigate biological processes among different pathway-associated patterns (17). GSVA enrichment analysis is a non-parametric and unsupervised method and could estimate the variation in pathway and biological process activity based on an RNA-seq expression dataset (17). We run this GSVA analysis using gene sets of “c2.cp.kegg.v6.2.symbols,” which were downloaded from the MSigDB database (18).

### Identification of Representative Genes by WGCNA

Using WGCNA (19), we could obtain the highly coexpressed gene modules, which were considered as the specific markers of phenotype in the tumor. Based on RNA-seq of GEP-NENs, the robust quantification of the absolute abundance of eight immune and two stroma cell populations in tumor tissues was calculated by the MCP-counter package of R software (20). In our study, plenty of GEP-NEN samples drove us to use the WGCNA method to identify the specific cell markers including these immune or stromal cells. We merged those modules with similar heights and increased module capacity with the cutoff threshold of <0.25. Gene significance (GS, the correlation between genes and cell fractions) and module membership (MM, the correlation between the genes and gene expression profiles of a module) were used to assess the relationships of all genes and specific phenotypes (immune or stroma cells). In our study, we identified representative markers in a module with the cutoff threshold of high MM and GS values (GS.cor >0.6 and MM.cor >0.7, respectively).

### Immune Cell Enrichment Analysis According to Single−Sample Gene−Set Enrichment Analysis Score

We quantify the infiltration level of 28 immune cell types using the single−sample gene−set enrichment analysis (ssGSEA) method, which provided the enrichment score to measure the abundance of immune cells. Gene set signatures of these immune cell types were obtained from a previous study (21). The ssGSEA score was normalized to unity distribution, and zero was the minimal and one the maximal score for each type of immune cell. Besides, the ssGSEA also involved T-cell activity, including T-cell coinhibition/costimulation, and type I/II IFN response. We also observed enrichment of parainflammation and inflammation-promoting gene expression profiles, and APC function, including MHC class I, HLA, and APC coinhibition/costimulation. Similarly, we also explored the subtypes of dendritic cells (DCs) including conventional DCs (cDCs), activated DCs (aDCs), plasmacytoid DCs (pDCs), and interdigitating DCs (iDCs). These gene sets are available in **Table S2**.

### Constructing the PC_Score and PI_Score Scoring System to Evaluate Individual GEP-NENs

1) The specific phenotype-related genes were identified. We used the specific pathway-related genes which had a strong correlation with cytolytic activity to conduct unsupervised hierarchical clustering. The DEGs between the subgroups were obtained using the limma R package. Only these DEGs which had a correlation to cell fractions with a threshold of MM >0.7 and GS >0.6 using WGCNA were used to conduct further exploration. We performed a univariate logistic regression model to calculate the odds radios of GEP-NEN occurrence. Then DEGs related to tumor occurrence were screened to construct a scoring system. 2) A scoring system was constructed. After obtaining the odds radios of each specific gene score, we then define the PC_Score and PI_Score of each patient: PC_Score/PI_Score = (β_i_ × Exp_i_),where i means the specific phenotype-related genes.

### Immunohistochemistry

The following antibodies were used for immunohistochemistry: SMARCA4 (ABclonal, Woburn, MA, USA, A19556), MLH1 (ABclonal, A20544), TSC1 (ABclonal, A0720), ATRX (Abcam, Cambridge, MA, USA, ab26457), and ATR (Proteintech, Wuhan, China, 19787-1-AP). The clinical tissue samples used in this study were histopathologically and clinically diagnosed at Fudan University Shanghai Cancer Center from 2014 to 2019. Two characteristics were used for scoring the expression of markers in slices: overall stain intensity (with possible values ranging from 0 to 3) and a score representing the percentage of tumor cells that were stained (1, 0%–25%; 2, 25%–50%; 3, 50%–75%, and 4, >75%). An immunohistochemistry (IHC) score was then calculated by multiplying the values of the two characteristics. Based on the IHC score, patients were divided into two groups: high expression (IHC score ≥median) and low expression (IHC score < median).

### Statistical Analysis

Spearman and distance correlation were used to calculate the correlation coefficient of these pathway-related molecules. The Wilcoxon test was used to compare the differences. To assess the specific gene associated with tumor occurrence, a univariate logistic regression model was used to calculate the odds ratio (OR). Because of the correlation between the four specific pathways and tumor occurrence, the “simDesign” package of R was used to identify the best cutoff point of each gene. All statistical analyses considered P < 0.05 as statistical significance.

## Result

### Cytolytic Activity in GEP-NEN Is Associated With Specific Signaling Pathways

**Figure 1:**
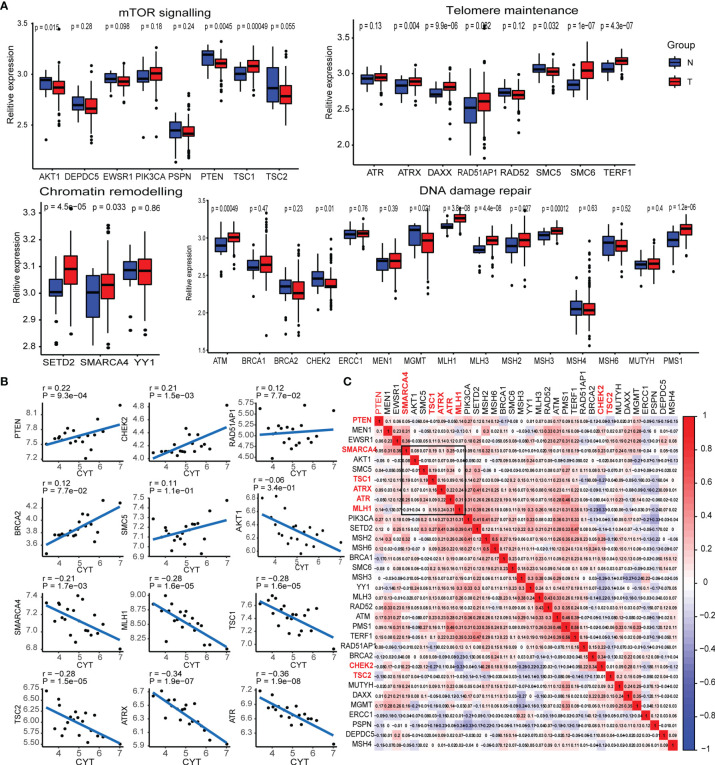
Transcriptional expression of the main pathway-related molecules in GEP-NENs. **(A)** Box plots show the expression distribution of 34-pathway-related molecules between paired normal (blue) and GEP-NEN (red) tissues. **(B)** Correlation between pathway-related molecules expression and cytolytic activity in GEP-NENs. **(C)** Correlation map of pathway-related molecules expression in GEP-NENs.

A previous study by the whole-genome landscape confirmed the occurrence of GEP-NENs involved in four main pathways: chromatin remodeling, DNA damage repair, activation of mTOR signaling, and telomere maintenance (6). Thus, we determined the expression level of majority of pathway-related molecules between paired normal and GEP-NEN samples. The results indicated that the expression of a majority of pathway-related molecules was significantly decreased in GEP-NENs (**Figure 1A**), which was consistent with a finding that most GEP−NENs had mutations in tumor-suppressor genes (22). However, we found that more molecules related to the process of chromatin remodeling and telomere maintenance were upregulated in GEP-NENs (**Figure 1A**). These findings illustrated the complexity of the carcinogenic mechanism of neuroendocrine tumors. Then, we explored if tumor cell-adjacent cytolytic activity was associated with specific pathway-related molecules. Importantly, we found that among the 34 common molecules associated with the four signaling pathways, only eight showed a positive and significant correlation with cytolytic activity (R^2^ >0.2, P < 0.001), named PTEN, CHEK2, SMARCA4, MLH1, TSC1, TSC2, ATRX, and ATR (**Figure 1B**). The coefficients of pathway-related molecules with cytolytic activity are available in **Table S3**.

Next, we assessed whether PTEN, CHEK2, SMARCA4, MLH1, TSC1, TSC2, ATRX, and ATR expression in GEP-NEN was coregulated or marked independently of GEP-NEN patients. An unsupervised hierarchical clustering of the RNA-seq expression revealed a significant correlation among expression levels of SMARCA4, MLH1, TSC1, ATRX, and ATR, while PTEN, TSC2, and CHEK2 did not show a significant correlation with the former gene cluster (**Figure 1C**). Of note, AKT1 and SMC5 also expressed a weak correlation with the gene cluster but was not included in our signatures because of the lack of a strong correlation with cytolytic activity (**Figure 1B**).

In order to verify the correlation of the expression of the specific pathway-related molecules (SMARCA4, MLH1, TSC1, ATRX, and ATR) with clinical information, we collected microarrays of pancreatic neuroendocrine tumors from 132 tissues, a total of 34 patients, including 30 adjacent tissues and 102 tumor tissues. We performed immune scores on these 132 tissues by IHC (**Figure S1**). We compared the immune score of these markers in adjacent tissues with that in tumor tissues, and the results indicated that the expressions of the specific pathway-related molecules were higher in adjacent tissue relative to tumor tissue (**Figure S2A**). However, when we analyzed the effect of these specific pathway-related molecules on relapse survival, the results indicated that these markers were not statistically significant to recurrence-free survival, but we also found that a high expression of some markers seemed to predict lower recurrence rates, such as ATR, MLH1, and SMARCA4 (**Figure S2B**). Interestingly, when we analyzed the effect of these specific pathway-related molecules on liver metastasis, we found that high expressions of ATR, ATRX, and SMARCA4 were associated with a low rate of liver metastasis (**Figure S2C**).

### Distinct Patterns of Specific Pathway-Related Molecules Associated With Cancer Hallmarks and Immune Infiltrations

We found the positive correlations among the five specific pathway-related molecules (**Figure 1C**) and found that the expression of pathway-related molecules was evenly distributed in the four main signaling pathways, but also a remarkable correlation was present among these molecules, respectively. Thus, the interplay among the four signaling pathways might be important for the generation of oncogenes or oncoproteins in GEP-NENs.

**Figure 2:**
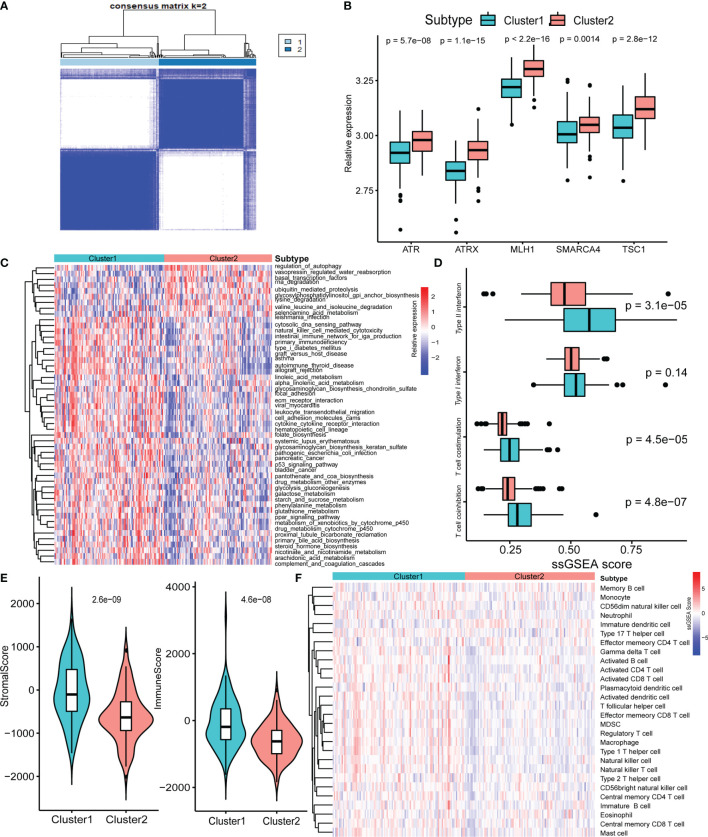
Biological characteristics of five-pathway-related molecules. **(A)** Unsupervised clustering of 5-pathway-related molecules. **(B)** The five-molecule expression level in the two-pathway-related molecules patterns. **(C)** Heatmap of GSVA enrichment analysis presents the activation state of biological signaling pathways in two-pathway-related molecules patterns. **(D)** Box plots of median ssGSEA scores of specific antitumor immune responses associate with T-cell activation between two-pathway-related molecule patterns. **(E)** Differences in the StromalScore and ImmuneScore between two-pathway-related molecule patterns. **(F)** Heatmap of ssGSEA scores of gene sets indicative of specific immune cell populations between two-pathway-related molecule patterns.

Next, we used consensus clustering based on RNA-seq expression of the five specific pathway-related molecules to classify patients with different specific molecules. After unsupervised clustering, 103 GEP-NEN samples from the five datasets were confirmed in cluster 1, while the other 101 samples were confirmed in cluster 2 (**Figure 2A**). Then, we compared the specific pathway-related molecules between them, and subtypes revealed higher expression levels in cluster 2 (**Figure 2B**). To identify the biological significance of these specific molecules, we found that cluster 1 was significantly enriched in carcinogenic activation signaling pathways, including p53 signaling pathways, ECM receptor pathways, and PPAR signaling pathways, and it also had a higher process in immune response including natural killer cell-mediated cytotoxicity, intestinal immune network for IgA production, and cytokine–cytokine receptor interaction (**Figure 2C**). However, cluster 2 was remarkably enriched in pathways related to cell proliferation and apoptosis, such as autophagy and RNA degradation (**Figure 2C**).

Plenty of studies had identified the relationship of immune or stromal cells with specific pathways (23, 24). Therefore, we attempted to analyze the potential function of specific pathway-related molecules in TME. Cluster 1 exhibited significantly higher enrichment of gene sets involving T-cell activity, including T-cell costimulation, and type I IFN expression (**Figure 2D**). Then, we used ESTIMATE packages, a method that uses gene expression signatures to infer microenvironment cell infiltration levels in tumor samples (25), to approximately measure the stroma and immune scores in GEP-NENs, and the result indicated that cluster 1 presented higher stroma and immune cell infiltration than did cluster 2 (**Figure 2E**). Prompted by these findings in the immune-related process between cluster 1 and cluster 2 in GEP-NENs, we next determined immune landscapes of the two patterns of pathway-related molecules. ssGSEA indicated a significant difference in the expression of markers for cytotoxic cells, in addition to an increased expression of marker gene sets for T-cell subsets including activated CD4 T cell, activated CD8 T cell, activated CD8 T cell, regulatory T cell, type 1 T helper cell, and type 2 T helper cell in cluster 1 (**Figure 2F**). Notably, activated dendritic cell, neutrophil, eosinophil, and B cell were also increased, suggesting the presence of the professional antigen-presenting immune process in cluster 1.

### Identification of Gene Modules and Gene Signature Correlated With Immune and Stromal Cells

**Figure 3:**
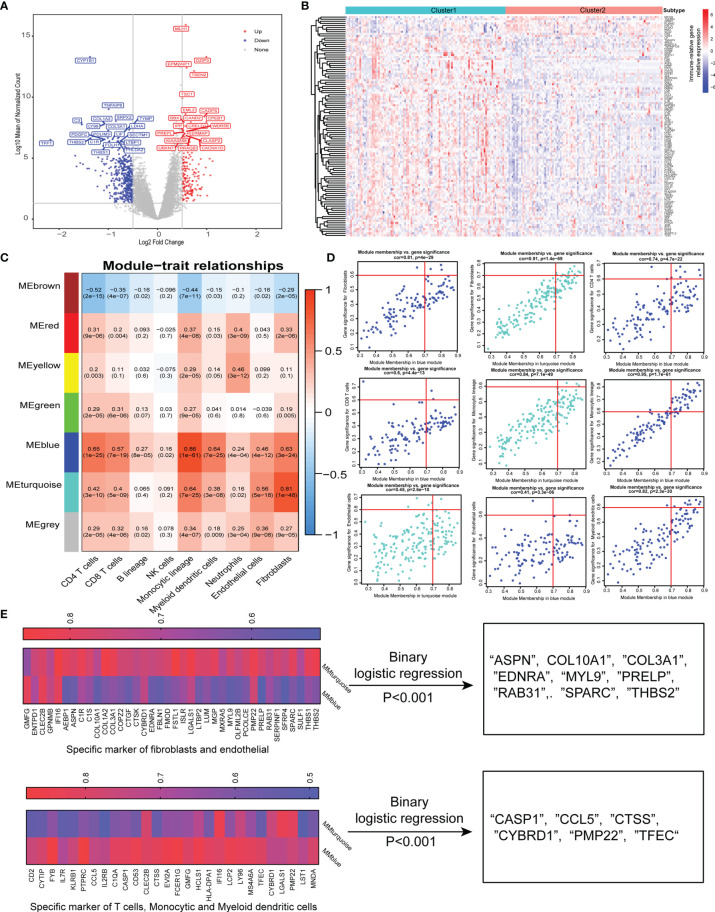
Identification of specific gene signature correlated with immune and stromal cells. **(A)** Differentially expressed genes between two-pathway-related molecule patterns. The expression of top 20 (red) and 20 (blue) upregulated genes in cluster 1 (blue) and cluster 2 (red). **(B)** Immune-related genes upregulated and downregulated in cluster 2. **(C)** Correlation between the traits and gene modules, including fractions of immune or stroma cell calculated by MCP-Counter. Correlation coefficients and P values are shown in each cell. The dendrogram on the left presents the degree of difference between the modules. **(D)** Genes with MM.cor > 0.7 and GS.cor > 0.6 were considered as specific markers for CAF and immune cells in turquoise and blue modules. **(E)** Canonical markers in turquoise and blue modules based on MM.cor and GS.cor calculated by WGCNA (left). The specific cell-related markers were screened by binary logistic regression (right).

To further characterize the functional role of the five specific molecules identified above, DGE analysis revealed 170 upregulated and 450 downregulated genes in cluster 2 compared with cluster 1 (**Figure 3A**). Interestingly, the top upregulated gene in cluster 1 was CYP1B1, which has been found to be associated with cancer development (26). Besides, TNFAIP6 was also found to be top upregulated in cluster 1, which was induced by proinflammatory cytokines such as TNFα and IL1. Then, we found a total of 105 immune-related DEGs while intersection was performed between 620 DGEs and immune-related gene sets published in the IMMPORT website (**Figure 3B**).

We used the MCP-counter package of R software to approximately calculate the infiltration of immune cell subsets and stromal cells including CAFs and endothelial cells, and 620 DEGs were used to effectively and objectively screen cell-specific gene sets by WGCNA methods. We chose β = 6 as a soft threshold to construct a scale-free network. The three models correlated with the CAF fraction; among them, blue and turquoise had a very close relationship in the cluster tree, which might originate from the identical type of cells. However, the red module was not homologous to them (**Figure 3C**). Interestingly, we also found that the blue and turquoise modules were associated with other immune cells including CD4 T cells, CD8 T cells, monocytic lineage, myeloid dendritic cells, and neutrophils. Under the conditions of GS.cor >0.6 and MM.cor >0.7, the gene signature or key genes mainly focus on blue and turquoise modules, and these modules are closely related to CD4 T cells, monocytic lineage, endothelial cells, and fibroblast cells (**Figure 3D**). These specific markers associated with immune and stromal cells in GEP-NENs are shown in **Figure 3E** (left). Then, in order to explore the specific markers associated with the occurrence and development of GEP-NENs, the binary logistic regression was conducted with the threshold of P < 0.001. Interestingly, the 9 specific markers were screened and all of them were associated with fibroblasts, including ASPN, COL10A1, COL3A1, EDNRA, MYL9, PRELP, RAB31, SPARC, and THBS2 (**Figure 3E**, right). Similarly, the 6 specific makers were screened, including CASP1, CCL5, CTSS, CYBRD1, PMP22, and TFEC, which focused on the CD4 T-cell and monocytic lineage (**Figure 3E**, right).

### Construction of Pathway-Related Subtypes Based on Specific Cell-Related Markers

**Figure 4:**
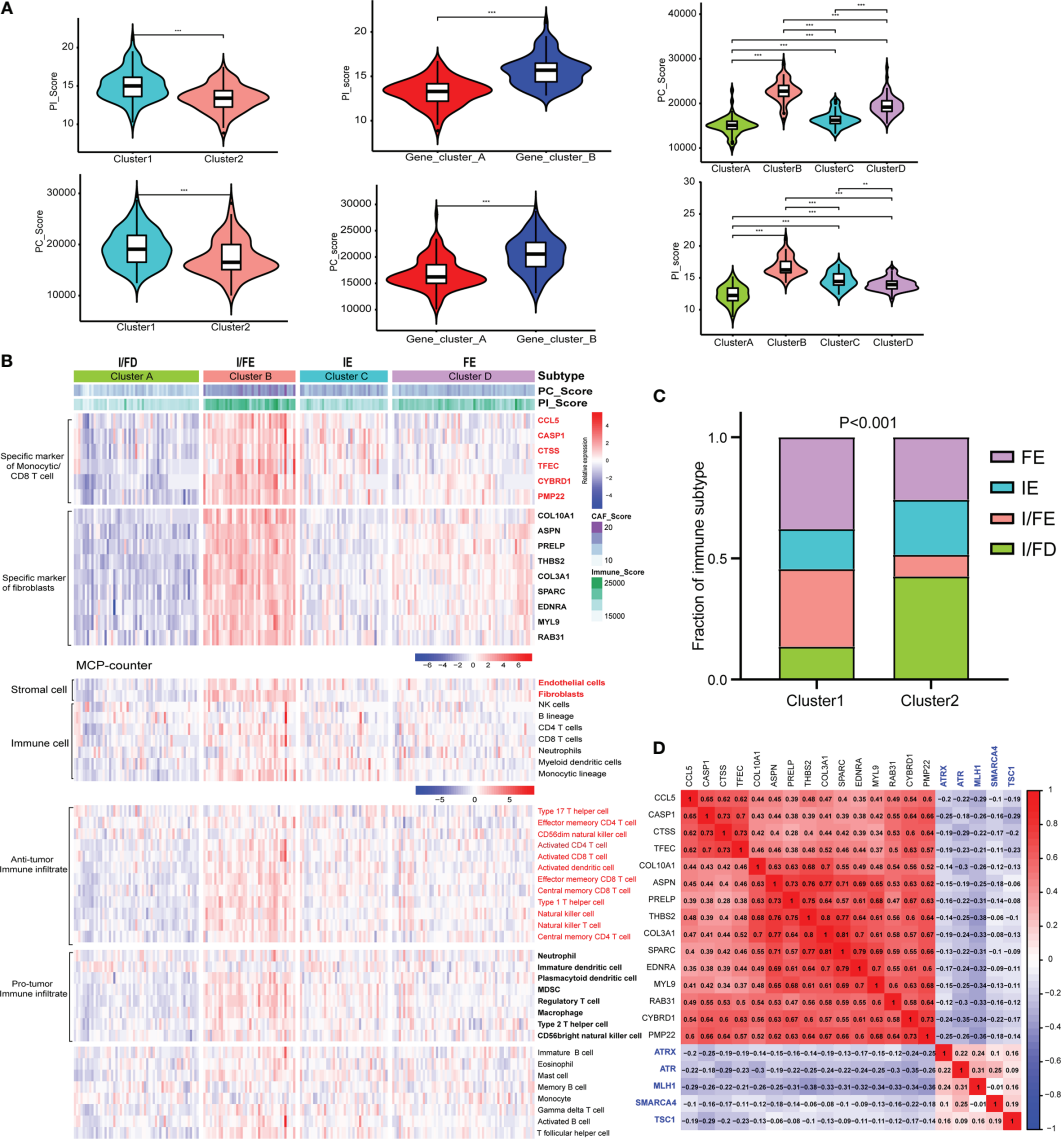
**(A)** Differences in the PI_Score/PC_Score between two-pathway-related molecule patterns (left). The differences in the PI_Score/PC_Score between gene_cluster_A and gene_cluster_B (middle). The differences in the PI_Score/PC_Score in the four subtypes based on the specific cell-related markers. **(B)** The heatmap to indicate the relative expression of specific markers among the four subtypes. Heatmap of ssGSEA scores of gene sets indicative of specific immune cell populations among the four subtypes. **(C)** The proportion of the four subtypes in the two patterns of pathway-related molecules. **(D)** Hierarchical clustering analysis was conducted by Pearson’s correlation coefficient between the cell-related genes and the five-pathway-related molecules. P values <0.05 were considered significant (ns, P > 0.05; *, P < 0.05; **, P < 0.005; ***, P < 0.001).

Given the heterogeneity and complexity of the four specific signaling pathways, we constructed a pathway-related score model using the specific cell-related markers to identify the microenvironment of individual patients with GEP-NENs. This model was defined as PC_Score and PI_Score (Specific Signaling Pathways CAF_Score and Immune_Score, see *Method*). The results indicated that PC_Score of cluster 1 was significantly higher than that of cluster 2, and similar results were also found in PI_Score. Interestingly, while we used the above 620 different genes between cluster 1 and cluster 2 to perform consensus clustering, the same two subgroups were also obtained, named gene_cluster_A and gene_cluster_B. gene_cluster_A had a significantly lower PC_Score and PI_Score than gene_cluster_B (**Figure 4A**). Then, we used the screened specific marker including 9 markers associated with CAFs and 6 makers associated with CD4 T-cell and monocytic lineage to unsupervised clustering, four subtypes in GEP-NENs were found, and we also compared PC_Score and PI_Score among them. The results indicated that cluster B was characteristic with the highest PC_Score and PI_Score compared with other clusters. In contrast, cluster A presents the lowest PC_Score and PI_Score. Another interesting result showed that cluster C presents a higher PI_Score relative to cluster D whereas cluster D has a higher PC_Score compared with cluster C (**Figure 4A**).

According to these novel findings, we composed a heatmap to indicate the relative expression of specific markers among the four subtypes (**Figure 4B**). Cluster B had the highest expression level while cluster A had the opposite trend. Cluster A and cluster B were designated as immune/fibroblast-enriched group (I/FE) and immune/fibroblast-deserted group (I/FD), respectively. Cluster C enriched with more immune specific markers was designed as immune-enriched group (IE), whereas cluster D with higher CAF-related markers were designed as CAF-enriched group (FE). Then, MCP-counter was used to explore the tumor environment among the four subgroups. I/FE presented the highest immune cell fraction and stroma cells including antitumor immune infiltration and pro-tumor immune infiltration, and I/FD was characteristic with the lowest immune and stromal cells (**Figure 4B**). Then, we explored the relationship of subgroups associated with CAF and immune cells with the two pathway-related patterns obtained from the 5 specific pathway-related molecules. Interestingly, cluster 1 consisted of a higher proportion of I/FE and FE, and cluster 2 was characteristic with a higher proportion of I/FD while the proportion of the IE group was not different from that of FE (**Figure 4C**). To explore the relationship among the specific CAF/immune cell markers and specific pathway-related markers, we calculated the pairwise correlations among the expression of 15-cell-related markers and 5-pathway-related markers and found that negative correlations were found between them (**Figure 4D**). We further explored the signaling pathways characteristic of these different subgroups. The signaling pathways activated in the I/FE group were PPAR, P53, Hedgehog, and JAK/STAT signaling pathways (**Figure S3A**). However, the Notch pathways were found to be highest in the FE group, and mTOR signaling did not present to be significantly distinct among the four subgroups. The glycolysis gluconeogenesis pathways were also higher in I/FE (**Figure S3B**), while citrate_cycle_tca_cycle and fatty_acid_metabolism did not present a distinct difference among these subgroups. Interestingly, tyrosine_metabolism was found to be highest in FE, while the I/FE group was characteristic with the lowest level of selenoamino_acid_metabolism, and I/FD had the opposite trend (**Figure S3B**).

### Molecular Subtypes Associated With Distinct Immune Cell Subsets

**Figure 5:**
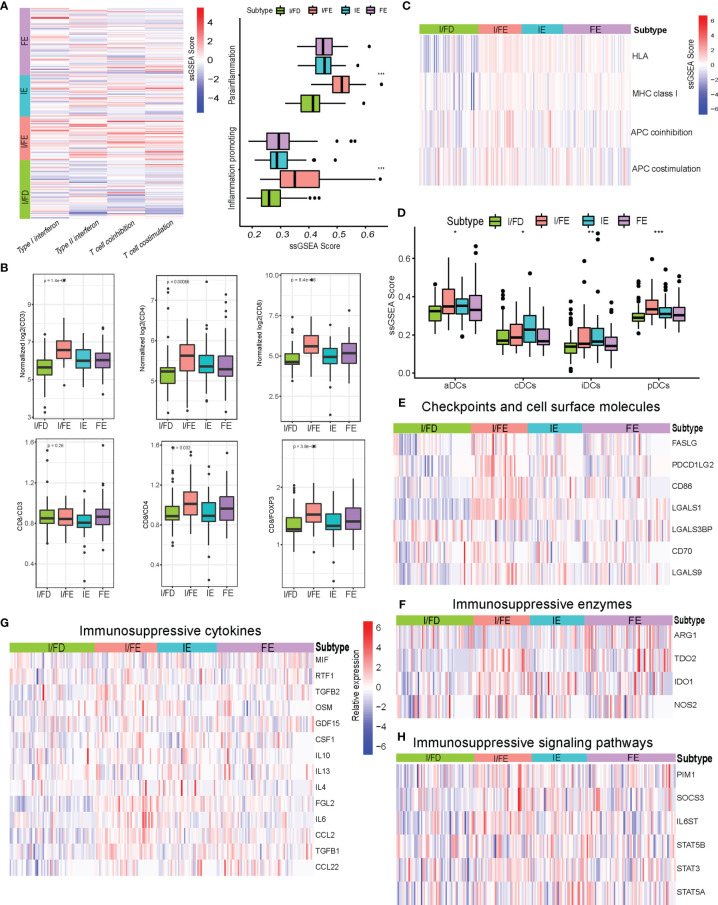
**(A)** Heatmap of median ssGSEA scores of specific antitumor immune responses associated with T-cell activation among the four subtypes. ssGSEA scores of two inflammation signatures (parainflammation and inflammation promoting). **(B)** Total CD3, CD4, and CD8, and the ratios of CD8/CD3, CD8/CD4, and CD8/FOXP3 to each for available matched samples respectively among the four subtypes. **(C)** Heat map of median ssGSEA scores of gene signatures upregulated in specific antitumor immune responses related to APC activation among the four subgroups. **(D)** Box of median ssGSEA scores of DC including cDCs, iDCs, pDCs, and aDCs among the four subtypes. **(E)** The comparison of immunosuppressive checkpoints and cell surface molecules among the four subtypes. **(F)** The comparison of immunosuppressive enzymes among the four subtypes. **(G)** The comparison of immunosuppressive cytokines among the four subtypes. **(H)** The comparison of immunosuppressive signaling pathways among the four subtypes. P values <0.05 were considered significant (ns, P > 0.05; *, P < 0.05; **, P < 0.005; ***, P < 0.001).

I/FE exhibited significantly higher enrichment of gene signatures involved with T-cell activity, including T-cell costimulation and type I IFN response (**Figure 5A**). Importantly, we also observed the highest enrichment of parainflammation- and inflammation-promoting profiles in I/FE, which had been predictive of response to immunotherapy in tumor (27) (**Figure 5A**). We then found increased levels of CD8 T-cell infiltration in I/FE, along with a higher number of CD3 T cells (**Figure 5B**). Interestingly, we also found increased higher CD4 T cell counts in the I/FE group. The ratios of CD8/CD4 and CD8/FOXP3 had a similar trend, while that of CD8/CD3 did not present to be distinct. These findings suggested that these subtypes were not only identified with high CD8 infiltration but reflected increased lymphoid infiltrate required for active antitumor immunity (**Figure 5B**). To obtain a more detailed understanding of immune-activating and immunosuppressive responses, we collected a list of immunosuppressive genes from previous publications to compare their expression among the four subtypes (28–32). Interestingly, the vast majority of immunosuppressive enzymes, cytokines, checkpoint ligands, and cell surface molecules as well as signaling pathways were activated in the I/FE group while the I/FD group was characteristic with the lowest level of these immunosuppressive molecules (**Figures 5E–H**), which might indicate that immune activation always coexists with immune suppression in GEP-NENs.

The effective T-cell response was due in part to the presentation of antigens and priming of T cells by professional antigen-presenting cells. The ssGSEA score indicated increased expression of gene signatures related to APC function, including MHC class I, HLA, and APC coinhibition/costimulation in the I/FE group (**Figure 5C**). Furthermore, the I/FE group displayed increased expression of aDCs and pDCs, which indicated the presence of a critical APC for antigen cross-presentation and potentially effector T-cell recruitment (**Figure 5D**). I/FE presented the highest number of dendritic cells, including iDCs and cDCs. These data suggested that I/FE was associated with innate immune-sensing pathways in GEP-NENs, and APC stimulation in immune response was critical for effective T cells.

### Molecular Subtypes and PC/PI_Score Involved in Chemokine Regulation

**Figure 6:**
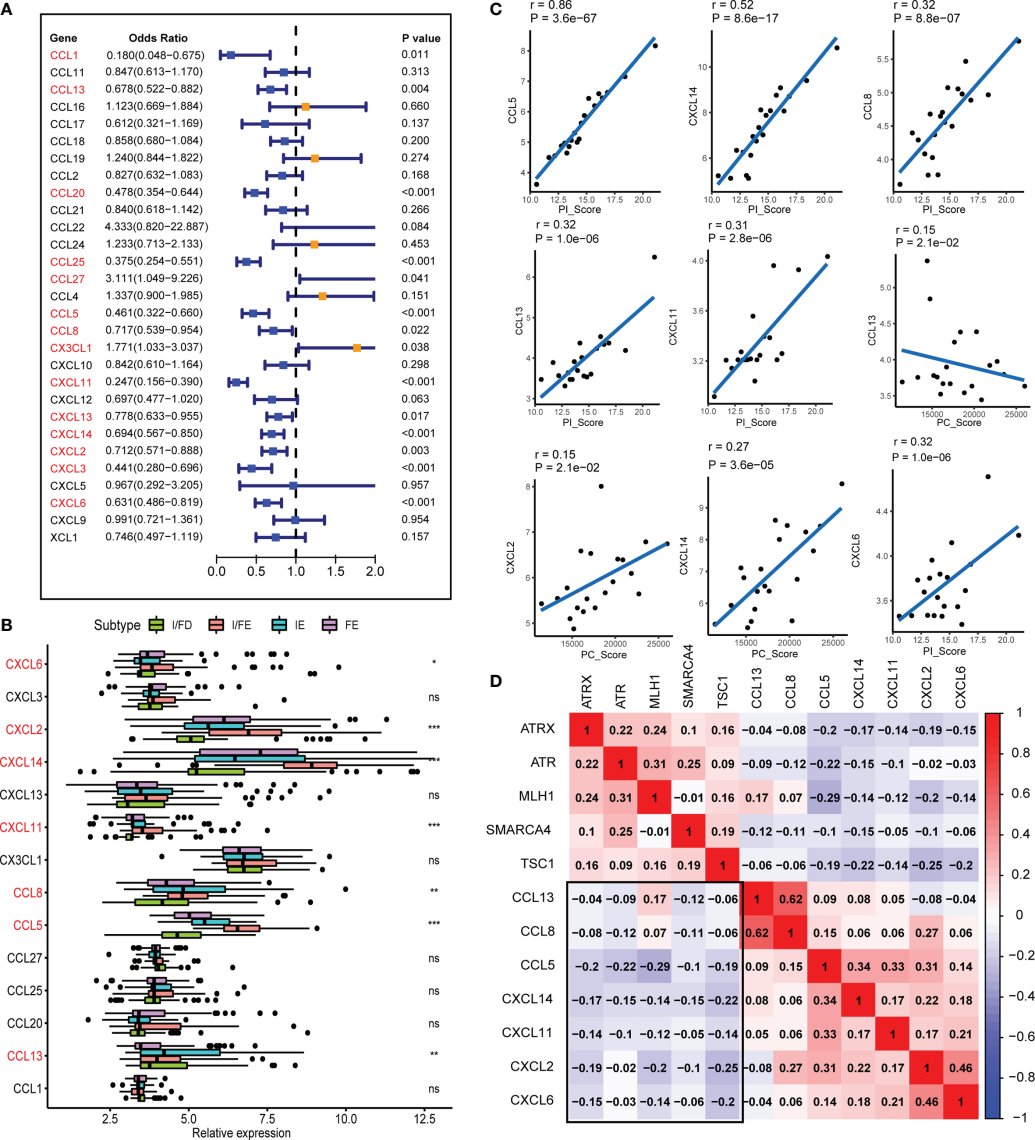
The chemokines associated with the four subtypes and PI_Score/PC_Score. **(A)** The univariate logistic regression analysis of 29 common expressed chemokines. **(B)** Differential expression of the 14 chemokines in normal and GEP-NEN tissues. **(C)** Pearson’s correlation analysis was used to assess the relationships between the PI_Score/PC_Score and the 7 key chemokines. **(D)** Hierarchical clustering analysis was performed using Pearson’s correlation coefficient between the 7 key chemokines and five-pathway-related molecules. P values <0.05 were considered significant (ns, P > 0.05; *, P < 0.05; **, P < 0.005; ***, P < 0.001).

Chemokines are chemoattractant cytokines that played a pivotal role in regulating the migration and infiltration of the immune cell population. More than 30 chemokines were acknowledged in GEP-NENs, each with its own specific pattern of cellular chemotaxis. To identify the chemokines associated with tumor occurrence and development in GEP-NENs, binary logistic regression was performed. About 14 chemokines were screened with the threshold of P < 0.05 (**Figure 6A**). Then, based on the 14 chemokines, we found that about 7 chemokines (CXCL6, CXCL2, CXCL14, CXCL11, CCL8, CCL5, and CCL13) were significantly correlated with the specific pathway-related subtypes (**Figure 6B**). Interestingly, most of these chemokines were highest in the I/FE group, while CCL13 presented a higher level in FE relative to the I/FE group, which was also consistent with these findings that high expression levels of CCL13 always coexisted with fibroblasts in tumors (33, 34). We also identified the correlations among the expression of 7 chemokines in GEP-NENs and found that positive correlations were more frequent than negative correlations with PC_Score and PI_Score (**Figure 6C**). These findings might suggest that the 7 chemokines played a critical role in the immune response and immune landscape among the four pathway-related subtypes. Besides, unsupervised hierarchical clustering of RNA-seq data revealed a significant correlation among expression levels of the 7 chemokines and the five specific pathway-related molecules, which also suggested that the 7 chemokines might directly or indirectly regulate the expression of pathway-related molecules, even immune landscapes (**Figure 6D**).

### Validation of These Findings in Other GEP-NEN Cohorts

Our findings had identified the expression levels of five-pathway-related molecules in GEP-NENs with a distinct T-cell phenotype and innate immune-sensing pathways. These results were tested in an independent replication cohort from 130 primary tumor (GSE98894). Consistent with the observations from the discovered cohort, the five-pathway-related molecules (SMARCA4, MLH1, TSC1, ATRX, and ATR) were correlated in the replication cohorts (**Figure S4**). The same four subgroups were also clustered based on specific cell-related markers, which exhibited distinct expression patterns, including cytotoxic cells, other T-cell population, and activated DCs, B cells, and neutrophils (**Figure S5A**). We also found that cluster 3 was characteristic with the highest PI_Score and PC_Score (**Figure S5B**). Furthermore, we observed the same increase in enrichment in parainflammation- and inflammation-promoting profiles predictive of immunotherapy efficacy in cluster 3 (**Figure S5F**), along with upregulation of T-cell or APC costimulation gene sets (**Figures S5C–E**). The vast majority of immunosuppressive cytokines, enzymes, checkpoint ligands, and cell surface molecules as well as signaling pathways were overexpressed in cluster 3 (**Figure S5G**).

### Liver Metastases Display the Distinct Subtypes Compared With Primary Sites

**Figure 7:**
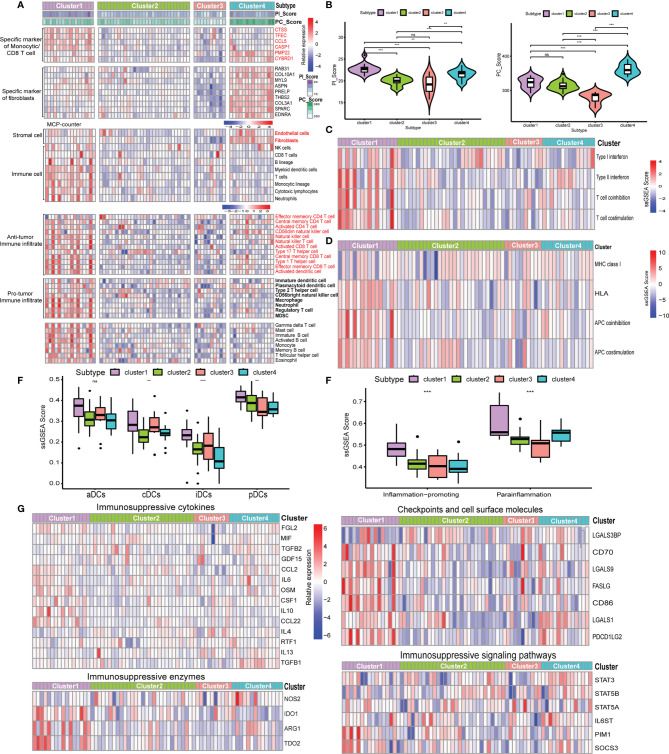
Expression of the five-pathway-related molecules was related to an effective immune landscape in 69 GEP-NENs liver metastases. **(A)** Heatmap of ssGSEA scores of specific antitumor immune cell populations using the same cell-related signatures. **(B)** The comparison of PI_Score and PC_Score among the four subtypes in liver metastases. **(C)** Heatmap of median ssGSEA scores of specific antitumor immune responses related to T-cell activation among the four subtypes. **(D)** Heatmap of median ssGSEA scores of gene signatures upregulated in specific antitumor immune responses related to APC activation among the four subtypes. **(E)** Box of median ssGSEA scores of DC including cDCs, iDCs, pDCs, and aDCs among the four subtypes. **(F)** ssGSEA scores of two inflammation signatures (parainflammation and inflammation promoting) among the four subtypes. **(G)** The comparison of immunosuppressive cytokines, enzymes, signaling pathways, checkpoints, and cell surface molecules among the four subtypes. P values <0.05 were considered significant (ns, P > 0.05; *, P < 0.05; **, P < 0.005; ***, P < 0.001).

The majority of GEP-NEN patients developed metastatic diseases, especially liver metastases. In the final set of analysis, we thus explored whether the relationship of the pathway-related subtypes and PC/PI_Score with an antitumor phenotype was conserved in GEP-NEN liver metastases. Therefore, we collected the GSE98894 gene sets including 69 liver metastases. The same gene signatures associated with CAFs and immune cells were conducted to perform unsupervised clustering. Notably, although the four subtypes could also be obtained, the I/FE subtype found in the primary site was lacking in the metastasis site, whereas the I/FD group could also be found but it was divided into two subtypes, characteristic with higher I/FD and lower I/FD (**Figure 7A**). From **Figure 7B**, our conclusion was confirmed, because the PI_Score was highest in cluster A, whereas the PC_Score in cluster A did not show a higher level compared with cluster B and cluster D. In contrast, cluster D was characteristic with the highest PC_Score while its PI_Score was lower relative to cluster A. In addition, we found that the I/FE subtype was lacking in liver metastases. Gene sets indicative of T-cell activation or suppression, DC stimulation, and MHC class I presentation also had the same landscape to the primary site (**Figures 7C–F**). The similar immunosuppressive signatures were also found in the IE subtypes (**Figure 7G**). Taken together, these data suggested that the pathway-related subgroups were distinct between the primary site and liver metastases, and the primary site had a higher proportion of I/FE subgroup whereas the liver metastasis was lacking.

## Discussion

In this study, we showed the potential role of pathway-related molecules in establishing immune landscape in GEP-NENs. Our initial observation was that among the molecules associated with the four pathways, only SMARCA4, MLH1, TSC1, ATRX, and ATR were strongly associated with cytolytic activity and led us to discover that the coexpression of the five-pathway-related molecules identified both primary tumor and liver metastasis with immune landscapes. Although our study could only demonstrate the capacity of the pathway-related genes to divide the GEP-NENs by the immune landscape, it is likely no coincidence that all five-pathway-related genes had known functions in activating effective immunity. SMARCA4 was essential for immune response, and the presence of a SMARCA4 alteration may confer a worse outcome to immunotherapy among lung cancer (35). Immune checkpoint inhibitors were preferable to conventional chemotherapy for MLH1-negative cancer because of the strong association between MLH1 and MSI status (36, 37). In addition, ATRX and ATR alterations contributed to tumor growth and immune escape because ATRX or ATR alteration could induce downregulation of genes linked to differentiation of tumor cells and destabilization of the immune checkpoint by some signaling axis, such as ATR-CHK1 (38, 39). As for TSC1, it acted as an important checkpoint for maintaining immune homeostasis by regulating cell fate determination (40). However, the interplay of these pathway-related molecules associated with immune landscape had not been explored; it is important to indicate that these molecules were important in mediating immune landscapes in other common tumors. Thus, our findings of strong and exclusive coregulation among these specific pathway-related genes denoted that similar mechanisms of these genes might also operate in GEP-NENs.

Then, based on the pathway-related genes, we also calculated the specific cell-related molecules and we reclassified GEP-NEN samples into four subgroups (I/FE, IE, FE, I/FD) and established a new PI_Score and PC_Score scoring model. In the subgroups, we assessed the immune status of GEP-NENs by exploring three essential processes in the immunity process (41): first, a direct cell–cell interaction between activated effector immune cells and their correspond cells, such as CD8 T cells; second, inhibition of activated immune cells leading to immune evasion, such as activation of immunosuppressive cytokines, checkpoints, and enzymes; and third, professional APC-mediated presentation of antigen to T cells. Notably, the expression levels of specific cell-related molecules marked the activity level of all four of these processes in the primary site of GEP-NENs while the same subtypes were not found in the metastasis site with the lack of I/FE although the IE, FE, and I/FD subtypes were clustered. Concretely, we used the specific pathway-related genes which had a strong correlation with cytolytic activity to conduct unsupervised hierarchical clustering. Only in these DEGs were both the subgroups which had a correlation to cell fractions with thresholds of MM >0.7 and GS >0.6 using WGCNA used to conduct further exploration. We used a univariate logistic regression model to calculate the odds radios of GEP-NEN occurrence. Stratifying patients based on the specific cell-related molecules segregated the cohort into the four subgroups in the primary site of GEP-NENs (I/FE, IE, FE, I/FD), characterized with distinct expression levels involving T-cell function, T-cell stimulation, and activity. Third, the professional APC activation was related to the pathway-related subtypes, involved in DC activation, costimulation of APCs, and MHC class I presentation. The presence and activation of DCs were known as critical processes for the cross-presentation of antigen and recruitment of effector immune cell (42). Interestingly, TSC1 expression by DCs had been reported to preserve T-cell homeostasis and response (43). Notably, the previous study demonstrated the crucial role for TSC1-mTOR in metabolic programming of the homeostatic DCs for T-cell homeostasis and implicated metabolic-coupled epigenetic imprinting as a paradigm for DC specification (44). Supporting this, our results also identified that the expression of a DC gene set was distinct among the four subgroups. This indicated that DCs were involved in mounting or priming of the anticancer immunity response, in primary tumors and metastases of GEP-NENs. Their potential functional roles in recomposing immune landscapes including controlling APC-mediated antitumor immunity in GEP-NENs required further attention.

Our findings supported recent evidence that pathway-related molecules could drive T-cell infiltration and antitumor response, but the relationship of these genes with immunogenicity was still difficultly explored because of the lack of public data. Upregulation of oncogenic pathways playing critical role in immunosuppression had been suggested (45). For example, KRAS signaling had been reported to be predictive of response to immunotherapy in colon tumor and could construct an immunosuppressive environment in pancreatic cancer (46). In an orthotopic model of pancreatic cancer, oncogenic KRASG12D signaling pathways were shown to drive an immunosuppressive environment by GM-CSF-mediated recruitment of suppressive myeloid populations, which could affect the function of effector CD8 T cells (46). Interestingly, the KRAS signaling in our study had been found to be enriched in I/FE, which might indicate that aberrant signaling drove the immunosuppressive environment. In addition, other tumor intrinsic mechanisms might affect the expression level of specific cell-related molecule expression. Wnt/b-catenin was associated with reduced CCL4 expression, which inhibited subsequent DC and T-cell recruitment in melanoma (47). Remarkably, several tumor intrinsic pathways had been reported to inhibit the antitumor immunity (48). Even so, these findings warrant further investigation in GEP-NENs. In addition, if tumor-intrinsic pathways could affect antitumor immunity, it remained to be determined whether an antitumor phenotype was also conserved among different primary and metastasis sites. Indeed, a previous study had reported that the expression of this 4-chemokine signature was consistently indicative of a T-cell phenotype across primary pancreatic cancer and liver metastases (49). However, our findings present that the immune landscape determined by five specific molecules associated with four critical pathways was not conserved in GEP-NENs. It will be interesting to assess primary and metastatic tumors and whether subcolonal cells of primary tumors seed into distant metastatic sites. Spatial heterogeneity, an uneven distribution of genetically distinct tumor cell subpopulations within disease sites, might be more applicable to explaining our finding.

Together, this study showed that specific pathway-related molecules were associated with transcriptional and cellular metrics of immune landscapes including effective T-cell response and APC response processes in GEP-NENs, both of which were required for antitumor immunity and could potentially serve as predictors of response to immunotherapy. We hoped that this correlation in GEP-NENs could find out the potential role of these pathway-related molecules in recomposing antitumor immunity in GEP-NENs. Indeed, the five-pathway-related molecules in our panel had been confirmed to be associated with immune cell infiltration and response to immune-checkpoint blockade, which provided an indication for a therapeutic strategy. Our findings might also be applicable for advanced GEP-NENs including liver metastasis. Understanding the underlying immunobiology in GEP-NENs might establish a new method to drive effector immune cell infiltration into the periphery of tumor cells and help more patients apply immunotherapeutic strategies.

## Data Availability Statement

The datasets presented in this study can be found in online repositories. The names of the repository/repositories and accession number(s) can be found in the article/**Supplementary Material**.

## Author Contributions

SJ, XY and YQ designed the study. XL, HG, and XX developed the methodology. XL, HG, XX, ZY, WZ, FW, JC, YZ, and XC performed the data analysis. XL, HG, and XX drafted the manuscript. All authors contributed to the article and approved the submitted version.

## Funding

This study was funded by the National Natural Science Foundation of China (U21A20374), Shanghai Municipal Science and Technology Major Project (21JC1401500), Scientific Innovation Project of Shanghai Education Committee (2019-01-07-00-07-E00057), Clinical Research Plan of Shanghai Hospital Development Center (SHDC2020CR1006A), Xuhui District Artificial Intelligence Medical Hospital Cooperation Project (2021-011), Shanghai Municipal Science and Technology Commission (20ZR1471100), National Natural Science Foundation of China (Nos. 82141129,82173281, 82173282, 82172577, 82172948, 81972725, 81972250, 81871950), Commission of Health and Family Planning (2018YQ06), and Shanghai Municipal Science and Technology Commission (19QA1402100).

## Conflict of Interest

The authors declare that the research was conducted in the absence of any commercial or financial relationships that could be construed as a potential conflict of interest.

## Publisher’s Note

All claims expressed in this article are solely those of the authors and do not necessarily represent those of their affiliated organizations, or those of the publisher, the editors and the reviewers. Any product that may be evaluated in this article, or claim that may be made by its manufacturer, is not guaranteed or endorsed by the publisher.

## Supplementary Material

Supplementary Figure 1 The overall stain intensity of these markers (SMARCA4, MLH1, TSC1, ATRX, and ATR) include “strong”, “moderate”, “weak”, “negative”, and we score these intensity levels “3”, “2”, “1”, “0”, respectively.

Supplementary Figure 2 The relationship between IHC score of these markers (SMARCA4, MLH1, TSC1, ATRX, and ATR) with clinical data. **(A)** The expression of these markers between adjacent tissue with tumor tissue by IHC. **(B)** The relationship of expression of these markers with recurrence free survival. **(C)** The relationship of expression of these markers with live metastasis.

Supplementary Figure 3 The specific signaling pathways among the four subtypes. **(A)** Heatmap of median relative expression of the cancer-related pathways among the four subtypes. **(B)** Box of median relative expression of metabolic-related signaling pathways among the four subtypes.

Supplementary Figure 4 Correlation map of five pathways-related molecules expression in 130 validated primary samples.

Supplementary Figure 5 Expression of the five pathways-related molecules was associated with an active immune phenotype in an independent GEP-NENs cohort. **(A)** Heatmap of ssGSEA scores of gene sets characteristic of specific immune cell populations using the same cell-related signatures. **(B)** The comparison of PI_Score and PC_Score among the four subtypes in validated cohort. **(C)** Heatmap of median ssGSEA scores of specific antitumor immune responses associated with T cell activation among the four subtypes. **(D)** Heat map of median ssGSEA scores of gene signatures upregulated in specific antitumor immune responses related to APC activation among the four subgroups. **(E)** Box of median ssGSEA scores of DC including cDCs, iDCs, pDCs and aDCs among the four subtypes. **(F)** ssGSEA scores of two inflammation signatures (parainflammation and inflammation promoting). **(G)** The comparison of immunosuppressive cytokines, enzymes, signaling pathways, checkpoints and cell surface molecules among the four subtypes.
