# Supplementary figures and images for: The Interplay of Four Main Pathways Recomposes Immune Landscape in Primary and Metastatic Gastroenteropancreatic Neuroendocrine Tumors
Source: Front Oncol. 2022 May 18;12:808448. doi: 10.3389/fonc.2022.808448 (PMC9158120; doi:10.3389/fonc.2022.808448)

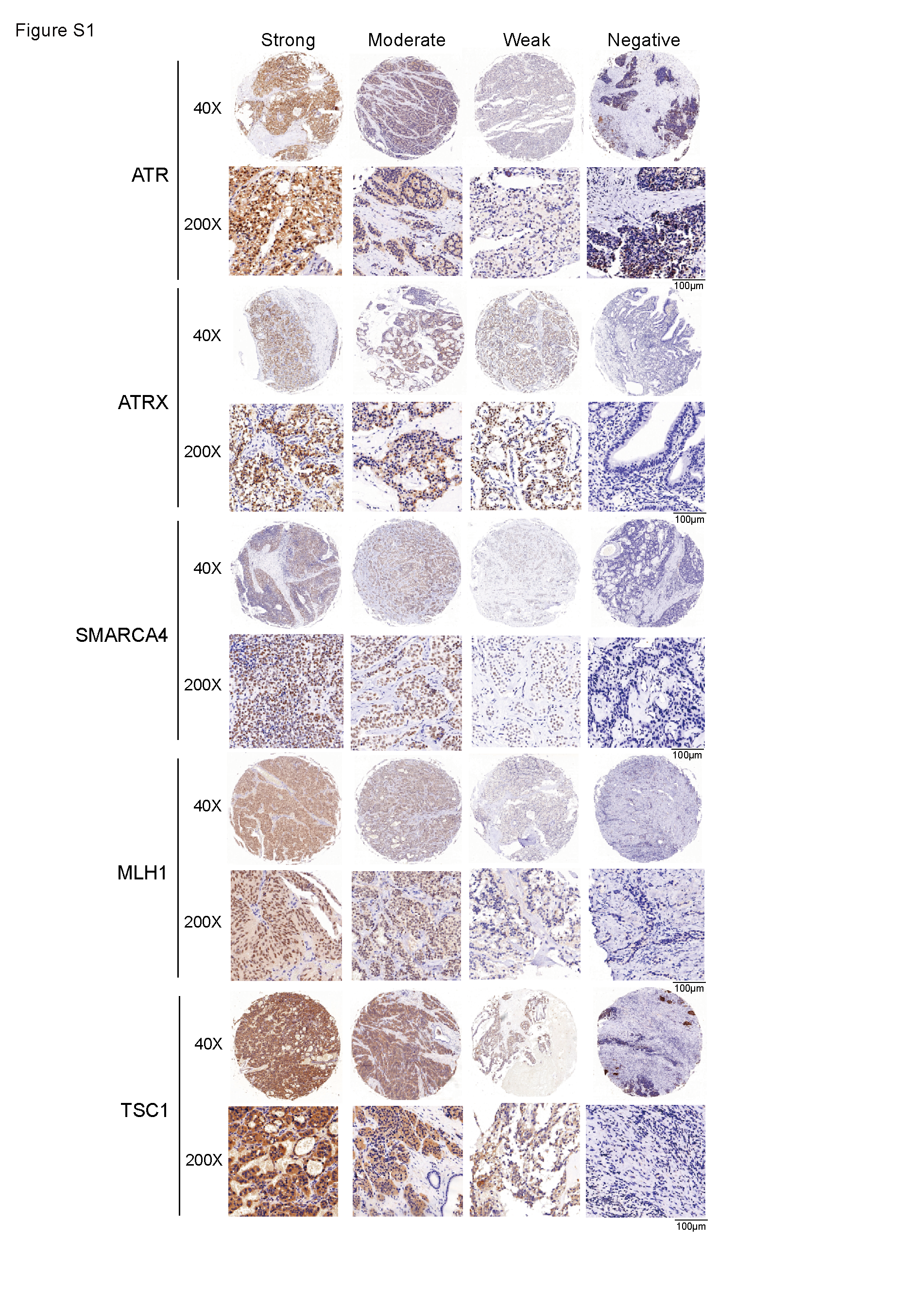

Supplement: Supplementary Figure 1 — The overall stain intensity of these markers (SMARCA4, MLH1, TSC1, ATRX, and ATR) include “strong”, “moderate”, “weak”, “negative”, and we score these intensity levels “3”, “2”, “1”, “0”, respectively. [file Image_1.tif]

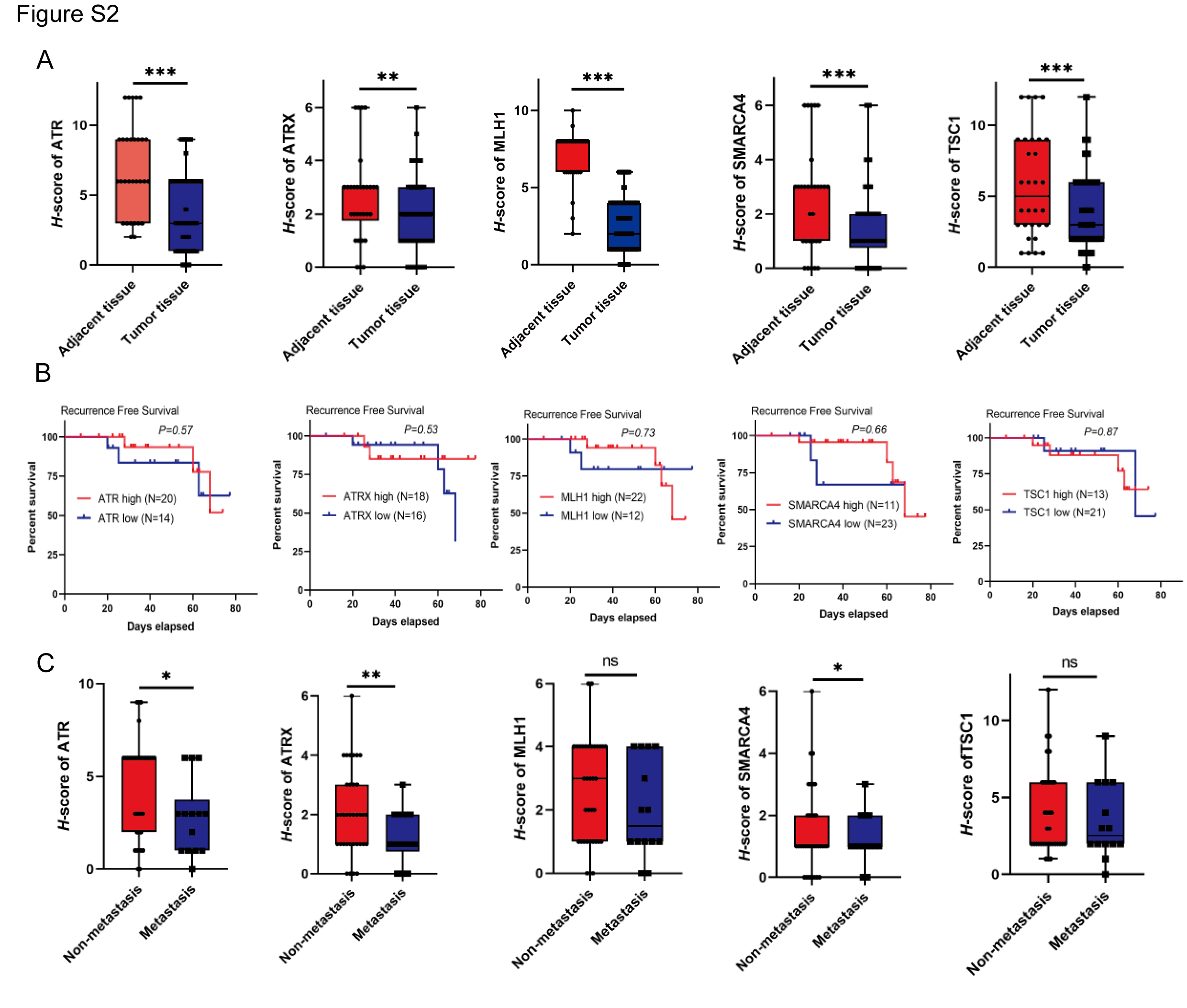

Supplement: Supplementary Figure 2 — The relationship between IHC score of these markers (SMARCA4, MLH1, TSC1, ATRX, and ATR) with clinical data. (A) The expression of these markers between adjacent tissue with tumor tissue by IHC. (B) The relationship of expression of these markers with recurrence free survival. (C) The relationship of expression of these markers with live metastasis. [file Image_2.tif]

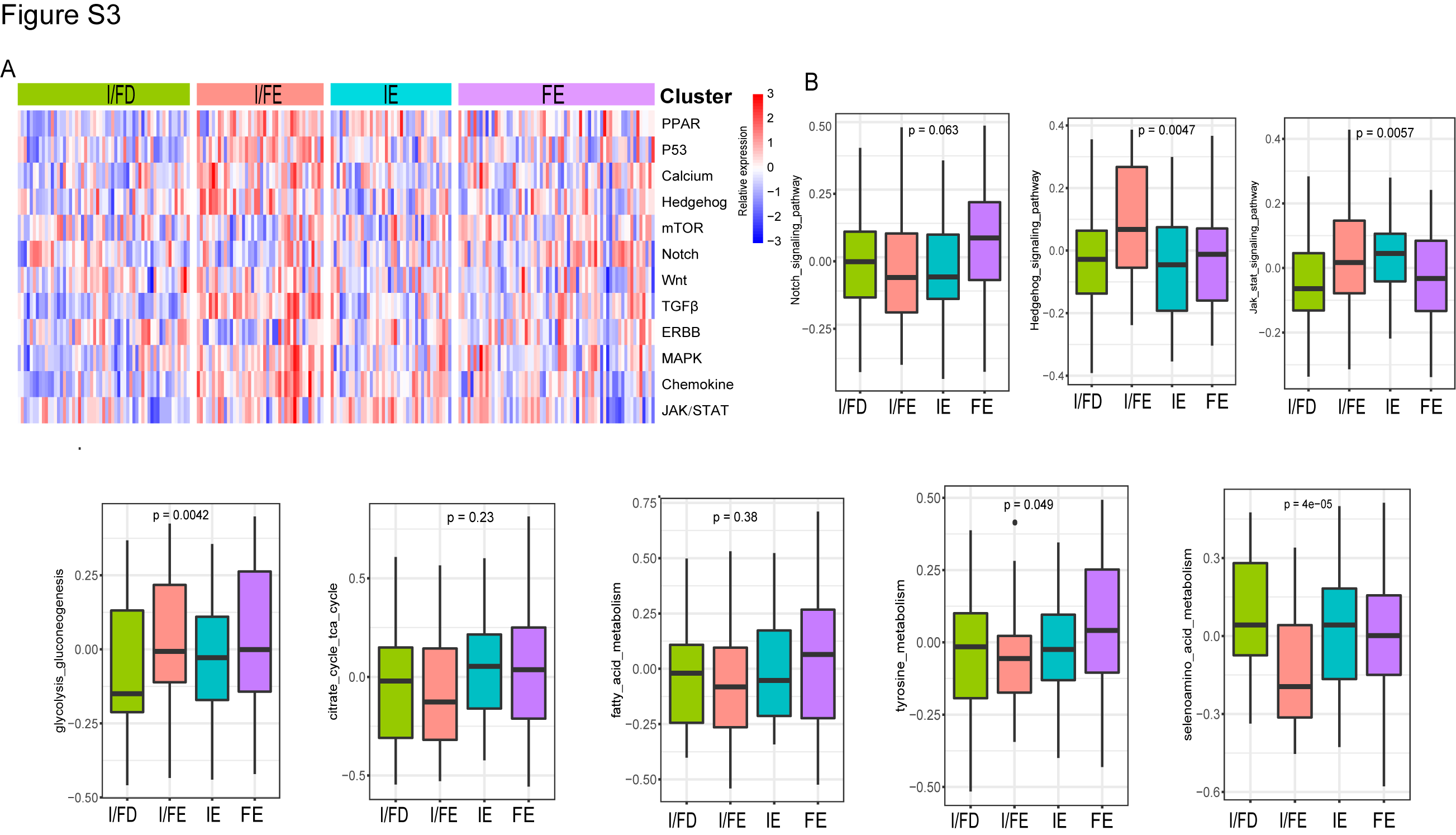

Supplement: Supplementary Figure 3 — The specific signaling pathways among the four subtypes. (A) Heatmap of median relative expression of the cancer-related pathways among the four subtypes. (B) Box of median relative expression of metabolic-related signaling pathways among the four subtypes. [file Image_3.tif]

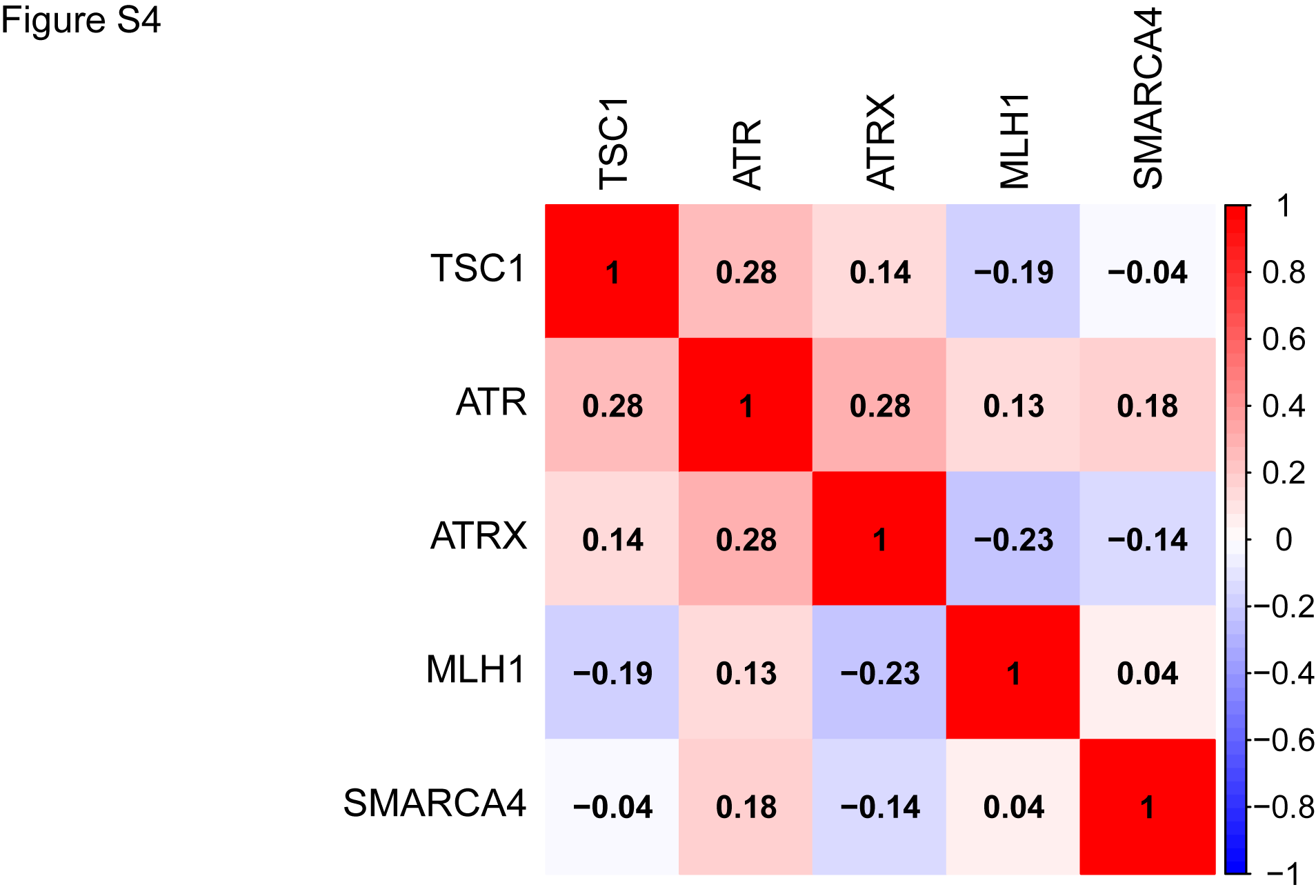

Supplement: Supplementary Figure 4 — Correlation map of five pathways-related molecules expression in 130 validated primary samples. [file Image_4.tif]

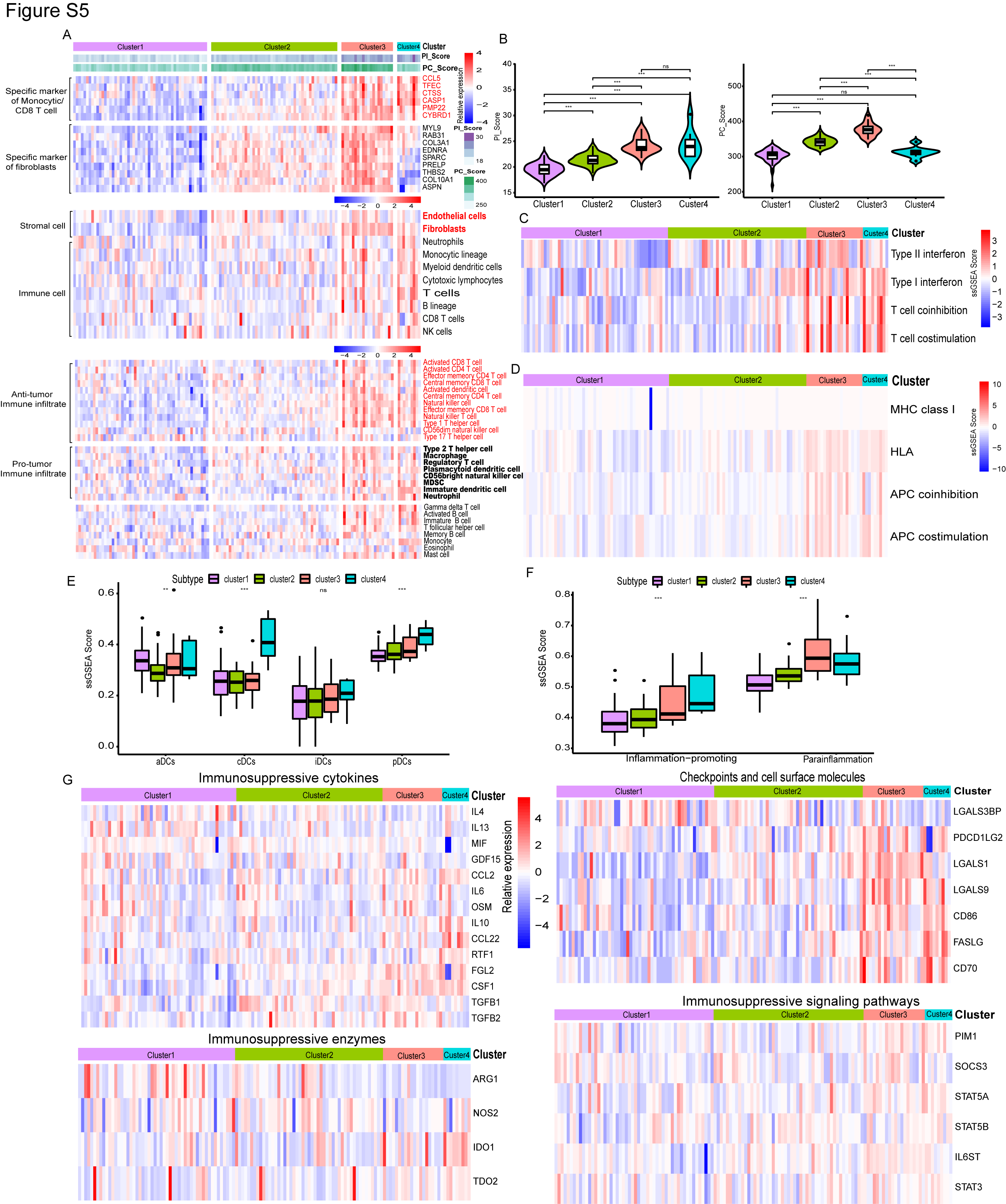

Supplement: Supplementary Figure 5 — Expression of the five pathways-related molecules was associated with an active immune phenotype in an independent GEP-NENs cohort. (A) Heatmap of ssGSEA scores of gene sets characteristic of specific immune cell populations using the same cell-related signatures. (B) The comparison of PI_Score and PC_Score among the four subtypes in validated cohort. (C) Heatmap of median ssGSEA scores of specific antitumor immune responses associated with T cell activation among the four subtypes. (D) Heat map of median ssGSEA scores of gene signatures upregulated in specific antitumor immune responses related to APC activation among the four subgroups. (E) Box of median ssGSEA scores of DC including cDCs, iDCs, pDCs and aDCs among the four subtypes. (F) ssGSEA scores of two inflammation signatures (parainflammation and inflammation promoting). (G) The comparison of immunosuppressive cytokines, enzymes, signaling pathways, checkpoints and cell surface molecules among the four subtypes. [file Image_5.tif]
